# A cross-sectional study exploring the relationship between regulator quality ratings and care home residents’ quality of life in England

**DOI:** 10.1186/s12955-019-1093-1

**Published:** 2019-01-31

**Authors:** Ann-Marie Towers, Sinead Palmer, Nick Smith, Grace Collins, Stephen Allan

**Affiliations:** Personal Social Services Research Unit (PSSRU), University of Kent, Canterbury, CT2 7NF UK

**Keywords:** Quality of life, Quality ratings, Care quality commission (CQC), National regulator, Adult social care outcomes toolkit (ASCOT), Care homes, Long-term care, Quality

## Abstract

**Background:**

The quality of life of people receiving health and social care is an important indicator of service quality, but the relationship between patient experience and outcomes and regulator quality ratings in England is unknown. In 2013, the health and social care regulator in England, the Care Quality Commission (CQC), introduced a new ratings system and by February 2017, all social care services were inspected and awarded new quality ratings (outstanding, good, requires improvement and inadequate). This study aimed to explore whether quality ratings were associated with residents’ quality of life, controlling for confounding variables.

**Methods:**

We conducted a nested, cross-sectional study, collecting social care-related quality of life (SCRQoL) data for 293 older care home residents in 34 care homes (20 nursing and 14 residential) in the South East of England. CQC ratings and other resident and home-level variables were also collected for the analysis. Multilevel modelling explored whether residents’ social care-related quality of life (SCRQoL) was associated with regulator ratings, controlling for confounding variables.

**Results:**

Outstanding and good homes were collapsed into one category and compared with homes requiring improvement. Nationally, only 2 % of care homes for older people are rated as inadequate and it was not possible to capture sufficient numbers for the analysis. We recruited one but it was re-inspected during the fieldwork period and its rating changed to requires improvement. The random intercept multilevel model, which accounted for 16.93% of the differences in SCRQoL within homes and 69.80% between, indicated that better SCRQoL was significantly associated with being female, better functioning, no dementia diagnosis, fewer communication difficulties, and living in a care home rated as outstanding/good by CQC. Size of home and registration category were not significant predictors.

**Conclusions:**

This study found evidence that quality ratings are associated with residents’ SCRQoL. As well as aiming to improve quality and ensure minimum standards, quality ratings have the potential to inform user choice and help the public compare care homes based on quality. Future research to establish the generalisability and replicability of the results is required.

## Background

Approximately 425,000 older people (age 65+) live in residential and nursing facilities (collectively called care homes) in England [1]. Many have moderate to advanced dementia [2], long-term health conditions and significant difficulties with activities of daily living [3]. The median life expectancy of a person moving into a care home in England is 15 months [4]. Thus, care homes are providing care and support for the most frail and vulnerable older people in our society. Ensuring the quality of this care is a subject that has attracted considerable media attention and the way it is measured and communicated to the public is an ongoing concern for the sector as a whole [5, 6].

English care homes operate in a quasi-market [7] with most providers being for-profit firms and only a quarter being not-for-profit or public sector [1]. The expectation is that market forces, such as competition between providers, should operate to drive up quality. However, research suggests that this is not necessarily the case, with competition between homes actually leading to reductions in fees paid by the public sector, which in turn drives down quality [8]. Contributing to this problem is the current economic climate facing local authorities in England. Unlike health care, long-term care (or social care, as it is called in England) is not free at the point of delivery. Local authorities have limited budgets for commissioning social care services (e.g. day services, domiciliary care, residential care) and have experienced consistent cuts over the last decade [9]. In response, some local authorities have had to reduce the prices paid to providers [10] and tighten their eligibility criteria for publicly funded service users [11]. Whilst approximately 50% of residents fund their own care [7], the degree to which they are able to ‘shop around’ is questionable [6] and they often pay over and above the fees being paid for publicly-funded residents [7]. Under these circumstances, regulation of care quality can play an important role in ensuring that quality is not consistently driven down or allowed to drop below basic minimum standards [7].

In England, the health and social care regulator, the Care Quality Commission (CQC), has recently undergone a period of transformation, moving from a system based on minimum standards to one of quality ratings [12]. Between October 2014 and February 2017, CQC inspected all adult social care services at least once [5], asking whether services are safe, effective, caring, responsive and well-led. Each question is graded as either outstanding, good, requires improvement or inadequate. The five separate ratings are then aggregated, according to an unpublished algorithm, into an overall quality rating using the same scale.

Prior to these changes, research had indicated that there were concerns amongst members of the public around how to judge the quality of services [6]. Research conducted in England, the Netherlands and Spain found that older people and their families particularly valued indicators representing residents’ quality of life [13]. In the research, several indicators were traded against one another and an aggregated measure of social care-related quality of life (SCRQoL), measured by the Adult Social Care Outcomes Toolkit (ASCOT) [14] was one of the three most valued. In England, ASCOT has also been piloted as an indicator of care home quality with local authority quality monitoring teams, who have an obligation to ensure the quality of the services they commission for publicly-funded people [15]. The ASCOT is a preference-weighted measure of SCRQoL developed to be sensitive to the impact of social care services. It has excellent face-validity [16] and has been found to be reliable, and associated with broader aspects of quality of life than health-related measures in older adults [17]. It is included in the Department of Health and Social Care's Adult Social Care Outcomes Framework (ASCOF) [18], as the overarching indicator for well-being, which all services should be aiming to improve [19].

Work undertaken when the Commission for Social Care Inspection (CSCI) was responsible for regulating and rating the quality of social care services, found that SCRQoL was related to quality star ratings for residential but not nursing homes, after controlling for individual and home-level characteristics [20]. The authors concluded that the focus of regulation at the time was on clinical and health processes, rather than social care outcomes, particularly in nursing homes [20, 21]. However, since then, the regulator and the quality ratings system has undergone significant reform and quality of life features more heavily in the five key lines of enquiry than it has in the past [22]. Indeed, the new regulatory model has at its core, a principle known as the ‘Mum test’ [5], which ask inspection teams to consider whether they would be happy for someone they love and care for to use the service. Given the public value placed on quality of life, and the expectation that the overarching CQC quality ratings will be used by the public to inform choice and enable the comparison of services based on quality, this study sought to explore whether residents living in homes rated as ‘good’ or ‘outstanding’ have better care-related quality of life than residents living in homes rated as ‘requires improvement’ or ‘inadequate’.

## Objectives

The objective of this study was to explore whether the new CQC quality ratings are associated with care home residents’ care-related quality of life, controlling for confounding home-level and individual-level factors.

## Methods

This study is reported using the Strengthening Reporting of Observational Studies in Epidemiology (STROBE) cross-sectional reporting guidelines [23].

### Study design and participants

The study employed a cross-sectional design, in which researchers spent 2 to 4 days in each home (depending on size and number of participants), carrying out observations and interviews. Additional data was collected via questionnaires completed by staff about the needs and characteristics of residents.

Power calculations (.80) to detect differences in the sample mean value of ASCOT of 0.05 or more from the hypothesised mean (mean = 0.69, SD = 0.25), using data collected in a national study of care homes [20], indicated that the study needed to achieve a minimum sample of 30 homes and between 210 and 340 care home residents.

In order to achieve the final sample of 34 homes (and a minimum target of 30 care homes), we employed an iterative recruitment process, selecting homes randomly from the publicly available CQC database of care homes, using a random number generator. Altogether, 119 homes were invited to take part, with higher numbers in initial waves reducing as more homes confirmed their participation. Only care homes for older adults, in two local authority areas in England, with a minimum of 10 residents, were eligible. An equal number of residential and nursing homes were invited during the initial two rounds of invitations, with further rounds targeting whichever type was lacking. The response rate was 29%, which is higher than previous research [24].

Within homes, managers were asked to coordinate resident recruitment with support from the researchers, randomly selecting residents from an alphabetical list and inviting them to take part in the research. Exclusion criteria was limited to temporary/short stay residents and those receiving palliative care. In accordance with the Mental Capacity Act [25] residents assessed as lacking the capacity to consent to take part in the research were recruited via the advice of a personal consultee. The Act defines a personal consultee as an unpaid carer or someone interested in the person’s welfare (such as a friend or relative), who is willing to be consulted. We asked home managers for advice on this and, where they felt consultees ought to be involved, they forwarded the appropriate information sheets and consent forms to consultees on our behalf. Alongside this, researchers spent time in each home talking to residents, explaining the study and assessing their capacity to consent. Throughout the study, researchers continuously monitored whether or not residents agreed to participate. Consent was considered a continuous process and researchers continuously assessed residents’ willingness to be involved in the study. This approach was approved by the National Social Care Research Ethics Committee. Between five and 10 residents were recruited per home, representing approximately 13–25% of an average size home (average is 40 beds [1]). As some of the homes were very large (up to 120 beds), we recruited more residents in these homes to achieve equivalent proportions. All permanent residents were eligible to take part, including those lacking capacity to consent.

Homes were inspected and rated under the new quality ratings system during the study. Therefore, for analysis we used the inspection report closest to the dates of fieldwork. Inspection report dates varied between April 2015 and November 2017, and the time between CQC inspection visits and our fieldwork ranged from 24 months to 6 days. This was controlled for by entering time difference into the models as a confound, although it was not found to have a significant effect.

### Dependent variable

The care home version of the Adult Social Care Outcomes Toolkit (ASCOT) was used to collect information on each resident’s social care related quality of life (SCRQoL). SCRQoL is conceptualised by eight domains (described in Table 1), found to be sensitive to social care interventions and services.

**Table 1:** The ASCOT domains

Domain	Definition
Control over daily life	The service user can choose what to do and when to do it, having control over his/her daily life and activities
Personal cleanliness and comfort	The service user feels he/she is personally clean and comfortable and looks presentable or, at best, is dressed and groomed in a way that reflects his/her personal preferences
Food and drink	The service user feels he/she has a nutritious, varied and culturally appropriate diet with enough food and drink he/she enjoys at regular and timely intervals
Personal safety	The service user feels safe and secure. This means being free from fear of abuse, falling or other physical harm
Social participation and involvement	The service user is content with their social situation, where social situation is taken to mean the sustenance of meaningful relationships with friends, family and feeling involved or part of a community should this be important to the service user
Occupation	The service user is sufficiently occupied in a range of meaningful activities whether it be formal employment, unpaid work, caring for others or leisure activities
Accommodation cleanliness and comfort	The service user feels their home environment, including all the rooms, is clean and comfortable
Dignity	The negative and positive psychological impact of support and care on the service user’s personal sense of significance

Most ASCOT measures are designed for self-completion (e.g questionnaires and interviews) and have response options reflecting four possible outcome states: ideal state, no (unmet) needs, some (unmet) needs and high (unmet) needs. As self-report is not a method accessible to many care home residents [26] and particularly those living in care homes, who often have moderate to advanced dementia [2], the care homes toolkit employs an inclusive methodology designed to give the resident as much opportunity to have a voice in their own quality of life ratings as possible, rather than relying solely on a single proxy-perspective. This is in line with the wider literature around the inclusion of people living with dementia in research, which notes that researchers have an ethical obligation to employ innovative and flexible research methods, which enable the inclusion of people with cognitive impairment in research [27–30].

The mixed-methods approach collects evidence about the eight domains through structured observations, structured or unstructured interviews with residents (where possible and at a level that gives them the greatest opportunity to engage with the topic), structured interviews with staff (always), and structured interviews with family members (where available). The researcher’s role is then to triangulate this evidence to make a rating in each domain. A full account of this mixed-methods approach is reported elsewhere [24]. Previous research involving this team has calculated inter-rater reliability (level of agreement of ratings made independently by researchers using the evidence collected) and found it to be good to excellent [24].

This study used a four-level version of this toolkit, which is conceptually equivalent to all the other ASCOT measures. Previously, the top two outcome states (ideal and no needs) in the care homes tool had been collapsed into one broad category indicating that the resident’s needs were met in each domain. However, as the ideal state is designed to reflect situations in which the care provided meets the individual’s wishes and preferences, we thought this was a useful distinction when exploring the relationship with regulator quality ratings. Authors two and four were the main raters for this study and inter-rater reliability is reported in the results.

Researcher ratings are weighted to reflect English population preferences [14] and entered into an algorithm (below) to calculate a score ranging from 1 to − 0.17 [14].

### Current SCRQoL = (0.203 x weighted score) – 0.466

Scores of one represent optimum or ‘ideal’ SCRQoL and scores of zero indicate a state that is equivalent, according to the preferences exhibited by the general population, to being dead. Negative scores indicate a state worse than being dead [14].

### Independent variables

#### Resident characteristics

We asked staff to complete questionnaires about participating residents’ functional abilities and levels of cognitive impairment, as well as demographic information, such as age, gender and so on. Questionnaires asked whether the resident had a diagnosis of dementia, their level of cognitive impairment, measured by the Minimum Dataset Cognitive Performance Scale (MDSCPS) [31], and their level of communication, as measured by the Dementia Communication Difficulties Scale (DCDS) [32]. This was included as there is evidence that homes find it more difficult to provide good quality of life for individuals with significant cognitive impairment [33] and because it is reasonable to expect that homes may struggle to meet the needs of residents who are unable to communicate them. Also requested was the amount of help residents required to carry out activities of daily living (ADL), such as mobilising, washing and dressing. These items have been used in previous research [34] and have been found to be associated with SCRQoL [20].

#### Care home variables

**Fig. 1:**
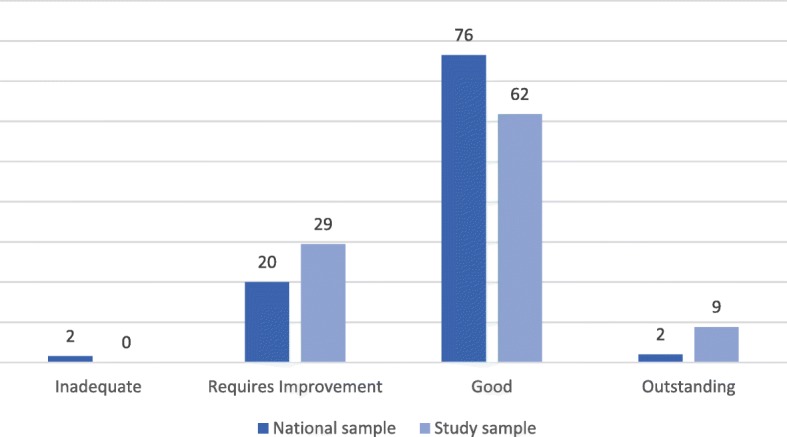
Percentage of homes in each CQC rating category for our sample and the national picture (Data for national ratings taken from The state of health care and adult social care in England report for 2016/17 [37]) Dark blue bars indicate the percentage of homes, nationally, with each quality rating (2% inadequate, 20% requires improvement, 76% good, 2% outstanding). Light blue bars show the percentages for the study sample (0% inadequate, 29% requires improvement, 62% good, 9% outstanding)

Contextual factors were collected about each home, including number of beds, registration (residential or nursing), sector (for-profit or not for-profit) and the local area’s income deprivation affecting older people index score (IDAOPI). The latter is the proportion of the area’s population aged 60 and over who are income deprived [35]. CQC ratings were recorded using the published reports for each home, from the new inspection regime. When a home was inspected more than once during the study, the inspection date closest to fieldwork dates was used. Possible CQC ratings include outstanding, good, requires improvement and inadequate [12]. One inadequate home was recruited to the study. However, as social care services rated as inadequate are placed into special measures and re-inspected within 6 months [36], this home was later re-rated as ‘requires improvement’. This second rating was now the closest in time to our own data collection, so the home was included in our sample as requires improvement. Only three (9%) homes in the sample were rated as outstanding (although this is proportionately more than the national picture of 2%), therefore outstanding and good were collapsed into one category. Figure 1 compares the distribution of homes nationally and within our sample for each quality rating. Data for the national ratings were taken from the State of Health Care and Adult Social Care in England report for 2016/17 [37].

### Statistical methods

To assess the degree that the two main raters provided consistency in their ratings across residents, inter-rater reliability was assessed using a two-way random, absolute agreement, single-measures Intraclass Correlation Coefficient (ICC) on the first 50 residents recruited to the study.

To investigate how the variance in resident SCRQoL was related to CQC ratings, it was also important to control for the other expected causes of variation at both resident and home-level. Therefore, a random intercept multilevel was run using the mixed model function in SPSS version 24 [38]. Due to the nested nature of the data, clustering was assessed using the ICC of the variance in SCRQoL due to differences between homes. ICCs between .05 and .20 are considered to indicate strong clustering of scores [39]. The ICC for this study was .139, so a multilevel model (MLM) was used.

The MLM was built in three stages, at each point checking for improvement in model fit. Full maximum likelihood estimation was used as it allows for the comparison of how well each model fits the data, by assessing the change in the − 2 Restricted Log Likelihood against the chi-square distribution of the associated degrees of freedom. Model one included only resident level variables, to control for the differences in resident characteristics between homes. Model two subsequently added the contextual variables, followed by model three, which included the main variable of interest, CQC rating.

SCRQoL scores were negatively skewed, as found in previous studies [20, 24, 40], however, examination of the residuals indicated that they were normally distributed and homoscedastic, with no autocorrelation (DW = 1.57). Therefore, the assumptions required for MLM were met, and no transformations were required [41]. Twenty-seven cases were excluded from the MLM, due to missing individual level data on the variables included in model 1. No homes were excluded.

## Results

### Descriptive statistics

Thirty-four care homes (20 nursing, 14 residential) across two local authorities in South East England took part in the study, between April 2016 and November 2017. The 34 care homes varied in size from 20 to 120 beds (M = 50.21, SD = 24.52). Seven homes (20.6%) were not-for-profit, 75% of which were good or outstanding, compared with only 66% of for-profit homes. This finding echoes previous research [7], however, it was not statistically significant. Although the number of not for-profit homes in the sample is representative of the national picture (18%), the low n may not be large enough to detect an effect. Therefore, sector was not included in further analyses. IDAOPI scores ranged from 0.05 to 0.35 (M = 0.13, SD = 0.07), indicating that participating homes were located in areas where the proportion of income deprived older adults was between 5 and 35% respectively.

The resident sample was 67% females and 33% males, with a mean age of 84.68 (SD = 8.66). The majority were widowed (52.6%), followed by married (23.5%), then single/never married (8.5%) or divorced (7.2%). Nearly all were white British (92.5%). The mean number of ADLs that residents could do independently was 3.57 (SD = 2.96; range 0–9). Around half were diagnosed with dementia (51.9%) and cognitive impairment across the sample ranged from intact (28%) to very severe impairment (6.5%), as measured by the MDSCPS. The mean score on the DCDS was 8.54 (SD = 9.11; range 0–39), with a higher score indicating more difficulty communicating.

### Controlling for differences between residential and nursing homes

Previous research indicates that levels of dependency and cognitive impairment are likely to be higher in nursing homes, and SCRQoL lower [20, 24]. Gender differences might also exist. Chi-squared tests showed that significantly more participants in nursing homes were male (X^2^ (1) = 4.08, *p* = .04), with no diagnosis of dementia (X^2^ (1) = 16.56, *p* < .05). Additionally, communication difficulties were significantly more prevalent (t(250) = − 2.22, *p* = .027). Despite differences at the national level [5], there was no difference in the dichotomised CQC ratings between nursing and residential homes in our sample.

### Social care-related quality of life (SCRQoL)

Based on ratings made for the first 50 care home residents recruited to the study, the resulting ICC for interrater reliability was excellent (ICC (2,2) = 0.88), suggesting only a small amount of measurement error was introduced by independent coders [42], and therefore statistical power in the subsequent analysis was not substantially reduced.

The mean current SCRQoL score of residents across all homes was 0.77 (SD = 0.16). As found previously, the sample is negatively skewed (skew = −.51, SE = .14) with scores ranging from 0.31 to 1.00. Sixteen residents (5.5%) had a score of 1.00, meaning that their SCRQoL was ideal across all eight domains.

**Fig. 2:**
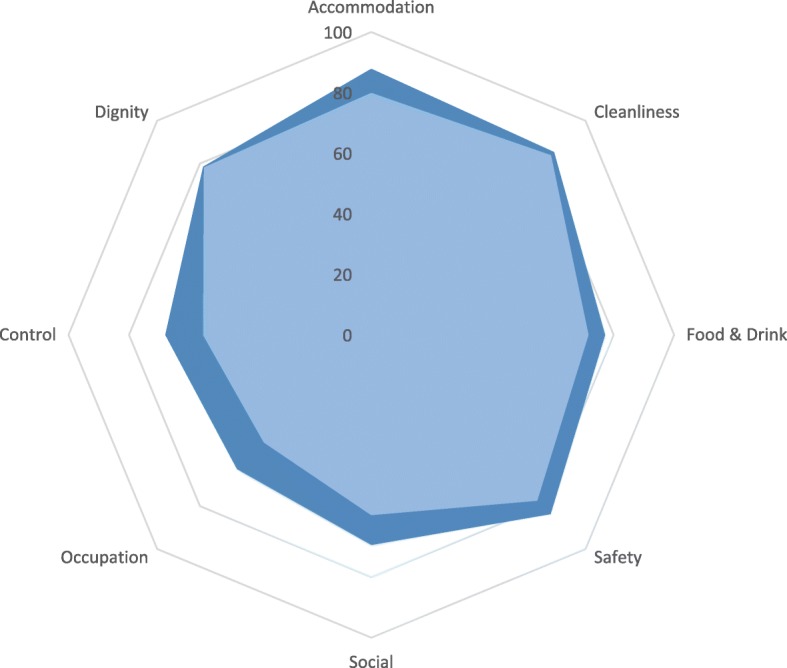
Cobweb plot comparing the average SCRQoL score in each domain as a percentage of the total possible score (unweighted). The dark blue shaded area represents outstanding/good homes and the light blue represents homes requiring improvement. The further out towards the edge of the plot the shading goes, the better the average score in each domain. 100% would mean that all residents had perfect scores in that domain (ideal state). 0% would mean that all residents had high (unmet) needs in that domain (worst possible score)

A comparison of the average domain scores (as a percentage of the maximum possible score in each domain) for homes rated outstanding/good and homes rated as requiring improvement is presented in Fig. 2. The overall shapes show that in all homes, regardless of quality rating, the higher order domains (control, occupation and social) revealed more unmet needs than the basic domains (accommodation, personal cleanliness, food and drink, and safety) [24, 43]. This is a pattern found in previous care home research in England [20, 24]. However, scores were lower in homes rated as requiring improvement, especially in accommodation, food and drink, safety, and the higher order domains of social participation, occupation and control over daily life.

### Care home quality

Ten homes in the sample had a CQC rating of requires improvement, and the remaining 24 homes were rated as either good or outstanding. These yielded a sample of 93 and 200 residents respectively. Tables 2, 3 and 4 show a comparison of the resident and home level characteristics respectively, at the different CQC ratings. Mean SCRQoL scores were significantly higher in homes with a good or outstanding rating, indicating that there is a relationship between care home quality ratings and resident quality of life. The only other variable which was significantly different between CQC ratings was diagnosis of dementia, where good or outstanding homes had higher numbers of residents living with dementia.

**Table 2:** Care homes quality ratings and relationships with resident characteristics

	Sample	CQC ‘Requires improvement’	CQC ‘Good’ or ‘Outstanding’	
Resident characteristics (*n* = 293)				
Female, n (%)	197 (67.2)	65 (69.9)	132 (66.0)	*X*^*2*^ = 0.44
				*p =* .51
Mean age (SD)	84.68 (8.66)	84 (8.30)	85 (8.85)	*t =* −0.35
min-max	50–103	62–99	50–103	*p =* .73
Mean no. independent ADLs (SD)	3.57 (3.00)	3.59 (3.13)	3.57 (2.88)	*t =* 0.05
min-max	0–9	0–9	0–9	*p =* .96
Diagnosis of dementia, n (%)	152 (51.9)	59 (63.4)	93 (46.5)	*X*^*2*^ = 5.03
				*p* = .03
Mean DCDS score (SD)	8.54 (9.11)	9.68 (9.00)	8.00 (9.13)	*t =* 1.43
min-max	0–38	0–38	0–38	*p =* .15
MDS CPS				*X*^*2*^ = 2.70
Intact, n (%)	82 (28.0)	22 (23.7)	60 (30.0)	*p =* .85
Borderline intact, n (%)	67 (22.9)	23 (24.7)	44 (22.0)	
Mild impairment, n (%)	28 (9.6)	9 (9.7)	19 (9.5)	
Moderate impairment, n (%)	33 (11.3)	13 (14.0)	20 (10.0)	
Mod severe impairment, n (%)	11 (3.8)	4 (4.3)	7 (3.5)	
Severe impairment, n (%)	24 (8.2)	9 (9.7)	15 (7.5)	
Very severe impairment, n (%)	19 (6.5)	5 (5.4)	14 (7.0)	
Total, n (%)	264 (90.1)	85 (91.4)	179 (89.5)	
Missing, n (%)	29 (9.9)	8 (8.6)	21 (10.5)	
Mean SCRQoL (SD)	0.77 (0.16)	0.71 (0.17)	0.79 (0.16)	*t =* −3.73
min-max	0.31–1.00	0.31–1.00	0.35–1.00	*p <* .001

**Table 3:** Care homes quality ratings and relationships with care home characteristics

	Sample	CQC ‘Requires improvement’	CQC ‘Good’ or ‘Outstanding’	
Home characteristics (*n* = 34)				
Mean no. of beds (SD)	50.21 (24.52)	58.57 (15.27)	52.41 (29.77)	*t =* 0.84
min-max	20–120	26–90	20–120	*p =* .41
Registered nursing, n (%)	20 (58.8)	6 (60.0)	14 (58.3)	*X*^*2*^ = 0.01
				*p* = .93
Mean IDAOPI score (SD)	0.13 (0.07)	0.12 (0.06)	0.13 (0.07)	*t =* −0.36
min-max	0.05–0.35	0.07–0.26	0.05–0.35	*p =* .73

**Table 4:** Multilevel regression models predicting Current SCRQoL

Parameters	Models					
	1 (*n* = 266)		2 (*n* = 266)		3 (*n* = 266)	
	Coef	SE	Coef	SE	Coef	SE
Intercept	.871^***^	.091	.880^***^	.101	.827^***^	.101
Resident variables						
Age	−.001	.001	−001	.001	−.001	.001
Gender	.040^*^	.019	.039^*^	.019	.041^*^	.019
ADL count	.009^**^	.003	.009^*^	.003	.009^**^	.003
Dementia diagnosis	−.062^**^	.022	−.064^**^	.023	−.064^**^	.023
DCDS	−.003^*^	.001	−.003^*^	.001	−.003^*^	.001
Home variables						
Registration			−.005	.028	−.006	.026
Number of beds			.000	.001	.000	.000
CQC rating					.073^**^	.024
Variance explained						
Within homes	17.23%		17.19%		16.93%	
Between homes	40.05%		41.01%		69.80%	
Model fit						
-2LL	− 275.167		−275.287		− 283.116	

*Note: † p < .10,* ^*^p < .05,^**^ p < .01,^***^p < .001. Gender coded as male = 0, female = 1; ADL count indicates number of ADLs able to do independently; Dementia diagnosis coded so that 1 = presence, 0 = no presence; Registration coded as residential home = 0, nursing home = 1; DCDS scored so that higher score = more difficulties communicating; CQC coded as ‘Outstanding’ or ‘Good’ = 1, ‘Requires improvement’ = 0

### Multilevel models

Table 4 shows the progression of the multilevel models. Model one included only resident-level variables, including age, gender, ADL count, dementia diagnosis and communication difficulties. This model accounted for 17.23% of the difference in SCRQoL within homes, where better SCRQoL was associated with being female, ability to do more ADLs independently, not having a diagnosis of dementia, and having fewer difficulties with communication. Age was not found to be a significant predictor.

Model two incorporated contextual home-level variables, however, neither number of beds nor home registration (residential or nursing) were significant predictors of SCRQoL. There was also no increase in the variance in SCRQoL explained between homes. IDAOPI scores were also initially added to model two, but were not significant and therefore removed in order to preserve the degrees of freedom at the home level.

When CQC rating was added however, it was found to be a significant predictor (B = .07, *p* = .006), with good or outstanding homes associated with better SCRQoL. This model accounted for 16.93% of the differences in SCRQoL within homes and 69.80% between, with a significantly better fit than the model with resident level variables only [Δ χ2(3) = 7.95, *p* < .05]. The effect size of a care home being rated outstanding/good on an individuals’ SCRQoL is 0.07. This is equivalent to 9.5% of average quality of life in the sample (95% CI: 3.3–15.8%). Thus, after allowing for both individual resident characteristics and contextual home level factors, SCRQoL was found to be significantly associated with CQC ratings.

## Discussion

This study is the first to explore the relationship between care home residents’ SCRQoL and the new CQC quality ratings [37]. Although not a national study, it was powered to detect differences in SCRQoL in this sample size and the model explained nearly 70% of the variance in SCRQoL between homes, which is very high. The results of the MLM indicate that residents living in outstanding/good homes have significantly better quality of life than those living in homes requiring improvement, after controlling for individual and home level characteristics. Although the effect size appears small, due to the scale upon which the scores are based, it is equivalent to 9.5% of average quality of life in the sample. It would be interesting to unpick the forces driving this improvement in quality of life in outstanding and good homes, however, we were unable to using the data available to us within this study. For example, it is possible that outstanding and good homes have less issues with the workforce, as an association has been found between staffing measures, such as retention and job vacancy rates, and CQC quality ratings [44]. Outstanding and good homes might invest more in staff training or have better staff to resident ratios [44]. We did try to collect information about ratios but had too much missing information to be able to include this in the analysis.

Another possibility is that outstanding and good homes have more activities or support to engage with activities and socialise with other people throughout the day but the evidence for this is somewhat mixed. For example, the overall pattern of results across the domains was very similar to that found in previous research in care homes [43], with more evidence of unmet needs in the higher order domains of control over daily life, occupation (how you spend your time) and social participation (spending time with people you like). Even in outstanding and good homes, quality of life was not as high in these domains as it was in the basic domains that are more traditionally catered for by social care services. The importance of these domains in adding quality to people’s lives should not be underestimated; they are the most highly valued in the preference weights, by the general population and social care users alike. As a population, we value the quality, not just the quantity, of years in our lives and the challenge for services is to find innovative ways of working with local communities to meet our expectations as we age, in a difficult financial climate. Unlike previous research conducted under a different regulator [20], we found that the relationship between quality ratings and quality of life of residents held for both nursing and residential care homes. This is interesting and is likely a reflection of the importance placed by CQC on quality of life in their key lines of enquiry. The social care-related quality of life domains included in the ASCOT can be mapped to some extent to all of CQC’s five key questions (are services safe, effective, caring, responsive to people’s needs and well-led?), with the question “are they effective” clearly having a focus on quality of life and resident outcomes. Given the broader move amongst policy makers to bring health and social care closer together, marked by the move towards greater integration of services and the recent name change of the Department of Health to the Department of Health and Social Care [45], this is a welcome finding.

Unfortunately, a limitation of this study was that we were unable to include any homes rated inadequate. Only 2% of homes nationally have this rating and they tend not to stay there very long as they are put into special measures for 6 months, making them harder to recruit and difficult to capture before they are re-inspected or closed. Another limitation is that we collected data in a relatively small sample size of care homes, all based in one region of England, and with nursing homes representing a larger proportion of the sample than nationally (59 to 37%). Related to this, the nursing homes in our sample had equivalent CQC quality ratings to the residential homes. Whereas, nationally, this is not the case, with nursing homes having poorer quality ratings [5]. Consequently, the generalisability of these findings is limited at this stage and results should be viewed as emerging evidence, requiring replication in future research.

However, one of the strengths of the research is that the dependent variable, SCRQoL, as measured by the ASCOT, is highly valued by older people and their relatives when comparing homes [13] and has been shown to be sensitive to the impact of social care interventions [14]. England does not have a minimum dataset for care homes, meaning that information about residents’ outcomes has to be collected through primary data collection, which is expensive and time consuming in a population who cannot self-report [26]. The mixed-methods approach enabled the study to include the most dependent care home residents, including those with dementia and communication impairments, who are the most at risk from low quality care but also have the greatest capacity to benefit from good care. It is promising that CQC quality ratings were associated with overall quality of life, as for many older people and their families this will be the most accessible information available when comparing homes.

## Conclusion

Commissioning high quality long-term care is a policy priority for many countries facing the challenges of an ageing population. In a climate of increasing needs and limited financial resources, there is an obligation to allocate resources wisely and support the most vulnerable older people in our society to live in high quality care facilities. This study provides a first look at the new CQC quality ratings in a sample of care homes for older people and found evidence that they are associated with residents’ care-related quality of life. This is important because, as well as aiming to drive up quality and ensure basic minimum standards, quality ratings have the potential to inform user choice and help the public compare care homes based on quality. Future research is needed to see whether these findings can be replicated on a national sample.
